# A Digital Closed-Loop Sense MEMS Disk Resonator Gyroscope Circuit Design Based on Integrated Analog Front-end

**DOI:** 10.3390/s20030687

**Published:** 2020-01-27

**Authors:** Yihang Wang, Qiang Fu, Yufeng Zhang, Wenbo Zhang, Dongliang Chen, Liang Yin, Xiaowei Liu

**Affiliations:** 1 MEMS center, Harbin Institute of Technology, Harbin 150001, China; 17B921023@stu.hit.edu.cn (Y.W.); 17B321006@stu.hit.edu.cn (W.Z.); zoom_chen@126.com (D.C.); liangyin.hit@gmail.com (L.Y.); 2 Key Laboratory of Micro-structures Manufacturing (Harbin Institute of Technology), Ministry of Education, Harbin 150001, China; 3 State Key Laboratory of Urban Water Resource & Environment, Harbin Institute of Technology, Harbin 150001, China

**Keywords:** disk resonator gyroscope (DRG), MEMS, digital control loop

## Abstract

A digital closed-loop system design of a microelectromechanical systems (MEMS) disk resonator gyroscope (DRG) is proposed in this paper. Vibration models with non-ideal factors are provided based on the structure characteristics and operation mode of the sensing element. The DRG operates in force balance mode with four control loops. A closed self-excited loop realizes stable vibration amplitude on the basis of peak detection technology and phase control loop. Force-to-rebalance technology is employed for the closed sense loop. A high-frequency carrier loaded on an anchor weakens the effect of parasitic capacitances coupling. The signal detected by the charge amplifier is demodulated and converted into a digital output for subsequent processing. Considering compatibility with digital circuits and output precision demands, a low passband sigma-delta (ΣΔ) analog-to-digital converter (ADC) is implemented with a 111.8dB signal-to-noise ratio (SNR). The analog front-end and digital closed self-excited loop is manufactured with a standard 0.35 µm complementary metal-oxide-semiconductor (CMOS) technology. The experimental results show a bias instability of 2.1 °/h and a nonlinearity of 0.035% over the ± 400° full-scale range.

## 1. Introduction

Since the last century, micro inertial sensors, especially the micro gyroscope, have found widespread developments in both research efforts and commercial products [1,2,3,4,5]. A gyroscope is a kind of inertial device for angular velocity measurement, mainly used in inertial navigation, industrial measurements, consumer electronics, etc. The solid wave gyroscope (SWG) was developed based on the solid wave theory proposed by Dr. David Lynch in the 1960s [6]. Compared with traditional vibration gyroscopes, SWGs have the advantages of higher precision, larger dynamic range, stronger anti-overload ability and better stability, which better fits the demands of inertial navigation [7,8,9,10,11]. However, the SWG represented by the hemispherical resonator gyroscope (HRG) has a large volume and is not suitable for mass production. These defects limit the use of SWG in tactical weapons and consumer electronics fields. On the other hand, with the rapid development of MEMS processing technology, various MEMS gyroscope were developed, and they rapidly occupied the market [12]. However, most of the traditional MEMS gyroscopes adopt a tuning fork structure, and cannot meet the requirements of high precision and stability. MEMS DRG is a combination of SWG and MEMS processing technology, with both of the advantages. MEMS DRG has become the research emphasis of high-performance MEMS gyroscopes.

Dr. David Lynchon proposed the principle of SWG on the basis of Bryan’s thin shell oscillator vibration theory. There are various types of SWG, which basically adopt an axisymmetric structure, including the HRG, ring resonator gyro, cylindrical resonator gyro, DRG and so on. Boeing first proposed the DRG design in 2006 [13], using a nested ring structure, consisting in a series of concentric rings connected through alternating spokes to a central mounting disk. The multiring design increases the electrodes’ area and effective mass, and provides high thermal stability and low anchor loss. At present, researches on contrapose DRGs are mainly supported by the micro-PNT (Micro-positioning, Navigation and Timing system) project of DARPA (Defense Advanced Research Projects Agency). Under the support of DARPA, the research on MEMS DRGs made a breakthrough. In a paper published in 2014 [14], Boeing achieved ultra-high performance with bias stability < 0.01°/h and ARW (Angle Random Walk) < 0.002/√h. This high performance attracted the attention to DRGs in the industry. In order to achieve high precision, the DRG has been optimized in many aspects, including design optimization of its sensitive structure [15,16,17], research on machining and manufacturing [18], working modes of DRG [19], control methods under full-angle mode [20,21], etc. Most of the research focused on the study of sensing elements and there are few reports on the design of the DRG readout circuit. Few studies mentioned the control circuits of DRG, and generally, the functions of DRG are verified by the combination of PCB discrete devices and FPGA (Field Programmable Gate Array) [22,23,24]. 

In this paper, a digital closed-loop sense MEMS DRG circuit design based on an integrated analog front-end is presented. Some innovations have been made in DRG readout circuit integration technology, quantitative analysis of the frequency splitting’s effect on DRG output and circuit implementation details. As mentioned above, the previous research on DRG readout circuits verified the functions of the sensing elements, without the consideration of integration and circuit implementation details. This design integrated the analog front-end and the self-excited drive loop on one chip for practical application. The model of the DRG’s mechanical structure is established to facilitate analysis and simulation. Force balance mode was adopted to realize the closed-loop sense, which satisfied high precision demands. A stable closed self-excited drive scheme with a high-frequency carrier is proposed, and sigma-delta modulation is used to convert signals. The ASIC (Application Specific Integrated Circuit) was manufactured in standard 0.35 µm complementary metal-oxide-semiconductor (CMOS) technology provided by HHGrace, the chip occupied 5mm × 5mm area. This work achieves a bias instability of 2.1 °/h with a full-scale range of ± 400 °/s.

This paper is organized as follows. Section 2 illustrates the mechanical structure modeling. System description and topology analysis are described in Section 3. Section 4 shows the circuit implementation details. Experimental results are shown and discussed in Section 5, and the conclusions are given in Section 6.

## 2. Mechanical Structure Modeling and Calculation

**Figure 1:**
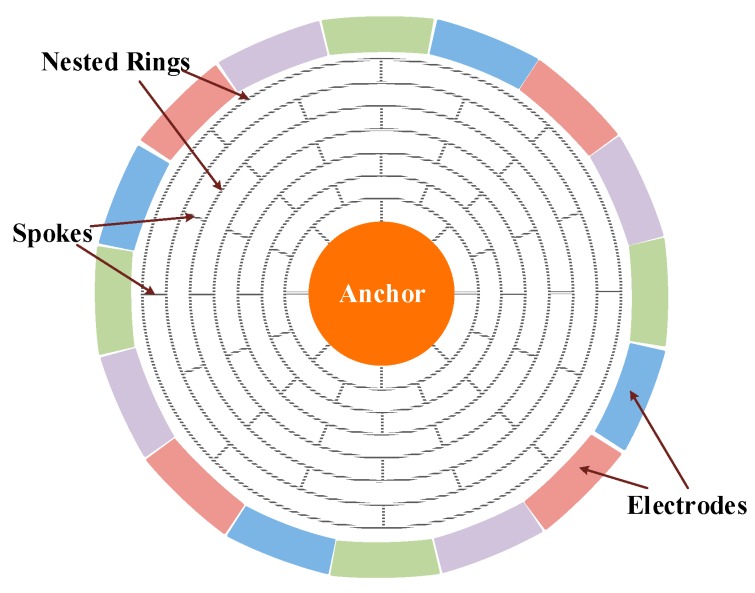
A standard model of the sensitive structure for the microelectromechanical systems disk resonator gyroscope (MEMS DRG).

Figure 1 shows the mechanical structure of the DRG. It has 16 evenly distributed discrete electrodes outside the shell, and inside the shell there are series of concentric rings connecting to the central anchor point through the alternating spokes. The fully symmetric structure can reduce the orthogonal coupling between drive mode and sense mode, and enable mode matching. The multi-ring design increases the electrodes’ area, increases the detection and drive capacitances and thus improves the detection sensitivity of the gyroscope. Meanwhile, the effective mass of the multi-ring design is increased and the influence of mechanical noise is reduced. 

According to the Coriolis principle, British scientist G.H. Bryan proposed the theory of standing wave precession in 1890. This theory indicates that the circular vibration is no longer stationary with respect to the shell but moves, when the oscillating axisymmetric shell rotates around the central axis. 

**Figure 2:**
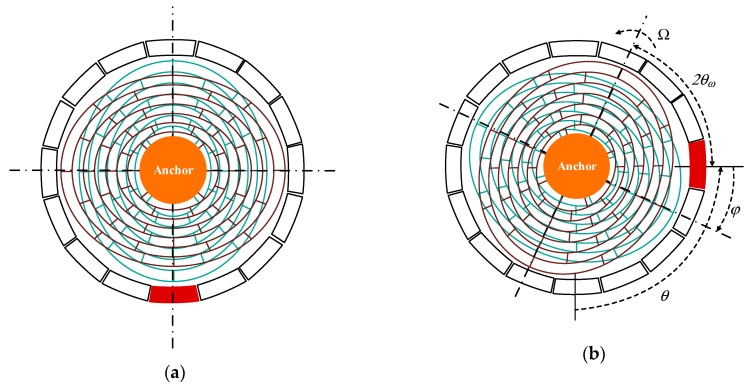
(**a**) Vibration mode of the stationary DRG, (**b**) Vibration mode of the rotating DRG.

Under the action of excitation, the resonator of the DRG maintains four antinodes of oscillation with circular wave number *n* = 2. In this state, the mode of resonator has four wave nodes and four wave antinodes, and in the form of standing wave. When the gyroscope is stationary, the positions of its wave nodes and antinodes remain unchanged, and the vibration mode is shown in Figure 2a. As it is shown in Figure 2b, the vibration mode generates annular precession relative to the shell under the action of the Coriolis force when the gyroscope rotates. In the second order vibration of the gyroscope, the input angular velocity of the gyroscope can be calculated by detecting the position of the vibration antinodes, or by detecting the applied voltage when the wave antinodes are kept in a fixed position.

There exists relative movement between the resonator vibration mode and the outside shell, when inputting angular velocity Ω. As the shell turns an angle *θ*, the vibration mode rotates angle *ϕ* relative to the shell. This angle is called the standing wave precession angle. The relationship between the two angles is shown in Equation (1)

(1) $$\theta =-K\varphi =-K\int_{0}^{t}\Omega dt$$

where, *K* is the precession factor. Equation (1) shows the linear relationship between *θ* and *ϕ*.

When the DRG operates under *n* = 2 mode, its motion can be equivalent to a simple resonance equation of two dimensions. The vibration radial displacement of each particle in time domain can be expressed by the following vibration mode function

(2) w(θ,t)=x(t)cosnθ+y(t)sinnθ

where, the *x*-axis is the drive mode direction, and *y*-axis is the sense mode direction, *x*(*t*) and *y*(*t*) express the displacement on the electrode axis of 0° and 45°, respectively.

The *n* = 2 vibration equation of DRG can be expressed as

(3) $$\begin{array}{l} m\ddot{x}-2nKm\Omega \dot{y}+b_{x}\dot{x}+k_{x}x=F_{x}\\ m\ddot{y}+2nKm\Omega \dot{x}+b_{y}\dot{y}+k_{y}y=F_{y} \end{array}$$

where, *K* is the precession factor and *ω*_0_ is the resonance frequency of DRG; *n* is the circular wave number; *b_x_* and *b_y_* express the resonator damping coefficients of corresponding axes, respectively; *k_x_* and *k_y_* are the resonator stiffness coefficients of corresponding axes, respectively; *F_x_* and *F_y_* represent external forces on corresponding axes respectively; *m* is the value of effective mass.

## 3. System Description and Topology Analysis

### 3.1. Working Modes of MEMS DRG and System Scheme Introduction

The working modes of DRG can be divided into full angle excitation mode and force balance mode according to different applications. The comparisons of these two working modes is discussed in Table 1. The dynamic performance of DRG under force balance mode is weak, but it can realize high sensitivity. This design focus is on low rotation speed and high precision applications. In view of this demand, force balance mode is adopted in this work. 

**Figure 3:**
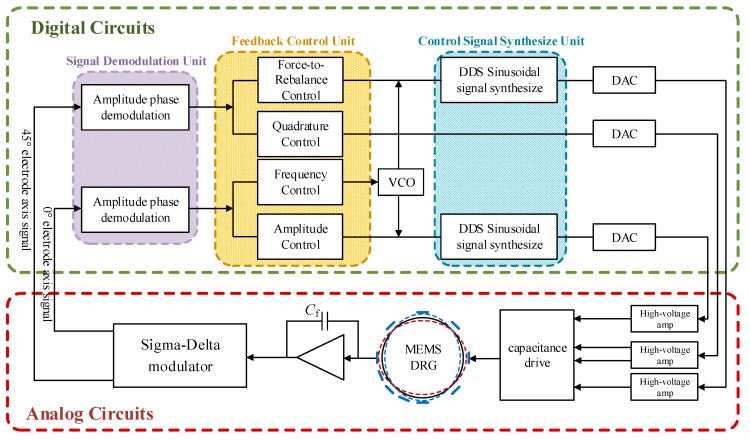
Overall scheme of DRG control circuit.

Comparing with vibration gyroscopes, DRG in this mode requires four control loops, which makes the system more difficult to design. The overall scheme of DRG control circuits is shown in the Figure 3. The signals of 0° and 45° axes is picked by sense electrodes, and the weak charge transforms on electrodes converts into voltage signal by the charge amplifier. This voltage signal is sent through a pre-filter into the analog to digital converter (ADC). Signal demodulation unit demodulate the sense signal to obtain its amplitude and phase. The feedback control unit needs four control loops. Frequency control to track the resonance frequency of DRG resonator. Amplitude control to maintain the amplitude of standing wave. Quadrature control and force-to-rebalance control to eliminate frequency splitting and control the azimuth angle of resonator vibration. In terms of function, the four circuits are respectively applied to the drive mode and sense mode of gyroscope. The control signal synthesize unit gives the digital control signals, these signals converted into analog signals through digital-to-analog converter and applied to the force electrodes. Through the above methods, this system can realize self-excited drive and closed-loop sense functions.

In the realization of control circuits, the analog control scheme has the advantages of low costs and high response speed, but analog circuits are complicated with low average accuracy, poor temperature characteristic and bad system reliability. In contrast, digital circuits have the advantages of high precision and reliability, easy system debugging and compensation, etc. This design adopts digital control methods, and combined the analog front-end and the digital closed self-excited loop on one chip. The circuit is divided into analog and digital parts as shown in Figure 3.

### 3.2. Study on Closed-Loop Self-Excited Drive

**Figure 4:**
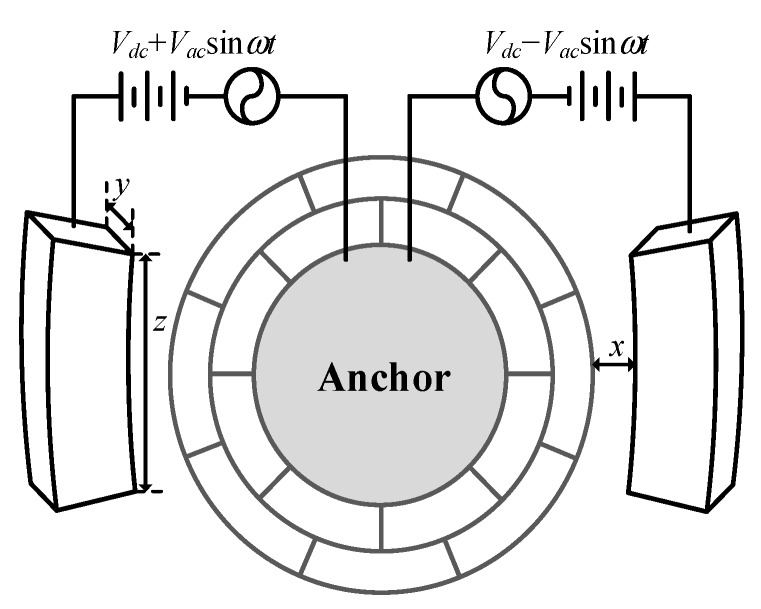
Infliction of the drive voltage.

The electrostatic force applied on external electrodes and anchor achieves the closed-loop self-excited drive. When a couple of alternating electrical signals are loaded between the drive electrodes and the anchor, the DRG is driven to vibrate along the direction of the drive force byelastic spokes connected to the concentric ring. Controlling the frequency of alternating signals allows gyroscope to resonate at its inherent frequency. The infliction of drive voltage is shown in Figure 4.

The resultant force on the drive direction can be obtained as followed when the drive electrodes are loaded with voltage value of *V_dc_* + *V_ac_*sin*ωt* and *V_dc_* − *V_ac_*sin*ωt* respectively

(4) $$\begin{array}{l} F_{tot}=N_{1}\varepsilon \left(-\frac{yz}{2x^{2}}\right)[{\left(V_{dc}+V_{ac}\sin\omega t\right)}^{2}-{\left(V_{dc}-V_{ac}\sin\omega t\right)}^{2}]\\ =-2N1\varepsilon \frac{yz}{x^{2}}V_{dc}V_{ac}\sin\omega t=F_{0}\sin\omega t \end{array}$$

where, *N*_1_ is the number of drive electrodes. In order to discuss the displacement dynamics equation in the drive direction, the influence of the sense direction can be ignored. According to Equation (3), the displacement can be obtained that

(5) $$\ddot{x}+\frac{b_{x}}{m}\dot{x}+\frac{k_{x}}{m}x=\frac{F_{0}\sin\omega t}{m}.$$

The general solution of Equation (5) is

(6) $$x\left(t\right)=\frac{F_{0}}{m\omega_{x}^{2}\sqrt{{\left(1-\frac{\omega^{2}}{\omega_{x}^{2}}\right)}^{2}+{\left(\frac{\omega }{Q\omega_{x}}\right)}^{2}}}\sin\left(\omega t+\arctan\frac{-\omega b_{x}}{m\left(\omega_{x}^{2}-\omega^{2}\right)}\right)$$

where, *Q* is the quality factor, $Q=\frac{m\omega_{x}}{b_{x}}$.

According to Equation (6), the DRG is subjected to simple harmonic vibration under the action of the periodic electrostatic force, when the frequency of the applied voltage is consistent with the inherent frequency of gyroscope. The closed-loop self-excited principle can be used to design the drive loop to obtain the same resonance frequency as the resonance frequency of gyroscope.

Based on the analyses above, a couple of differential alternating electrical signals are loaded to realize self-excited drive. Combining Equations (4) and (6), the vibration of DRG under differential alternation signals is

(7) $$x\left(t\right)=-2N_{1}\varepsilon \frac{yz}{x^{2}}V_{dc}V_{ac}/m\omega_{x}^{2}\sqrt{{\left(1-\frac{\omega^{2}}{\omega_{x}^{2}}\right)}^{2}+{\left(\frac{\omega }{Q\omega_{x}}\right)}^{2}}\sin(\omega t+\arctan\frac{-\omega b_{x}}{m\left(\omega_{x}^{2}-\omega^{2}\right)})$$

The frequency of alternating signal automatically tracks the resonance frequency of drive mode if closed-loop self-excited drive can be realized. Equation (7) can be simplified as

(8) $$x\left(t\right)=\left[2N_{1}\varepsilon \frac{yz}{x^{2}}QV_{dc}V_{ac}/m\omega_{x}^{2}\right]\cos(\omega_{x}t)$$

A corresponding current is generated on the detection electrodes of self-excitation when a DC voltage *V_drive_* is loaded on the central anchor. Supposing there are *N_2_* drive-sense electrodes, the total current is

(9) $$i=N_{2}V_{drive}\frac{dC}{dt}=N_{2}V_{drive}\frac{dC}{dx}\frac{dx}{dt}=-N_{2}V_{drive}\varepsilon \frac{yz}{x^{2}}\frac{dx}{dt}$$

Based on Equation (7), (9) is rearranged as

(10) $$i=-N_{2}V_{drive}\varepsilon \frac{yz}{x^{2}}\frac{dx}{dt}=\frac{2N_{1}N_{2}\varepsilon^{2}y^{2}z^{2}QV_{drive}V_{dc}V_{ac}}{x^{4}m\omega_{x}^{2}}\sin(\omega_{x}t)$$

The AGC (Automatic Gain Control) loop control adjust the amplitude of drive signal. The gain of AGC is *G* and the drive signal equals to

(11) $$V_{drive}=-\frac{2N_{1}N_{2}\varepsilon^{2}y^{2}z^{2}QV_{drive}V_{dc}V_{ac}}{C_{f}x^{4}m\omega_{x}^{2}}G\sin(\omega_{x}t)$$

where, *C_f_* is the value of charge amplifier feedback capacitance. According to the Barkhausen criteria, self-excitation is satisfied if it meets the condition of

(12) $$\frac{2N_{1}N_{2}\varepsilon^{2}y^{2}z^{2}QV_{drive}G}{C_{f}x^{4}m\omega_{x}^{2}}=1$$

### 3.3. Study on Sense Loop

In the practical system, numerous factors affect the vibration function. For instance, frequency splitting, asymmetric damping, mass imbalance, etc. Equation (3) is not sufficient to fully represent the vibration state of the resonator. Improved from Lynch’s model, the two-dimensional vibration equation of the DRG with error terms is obtained under non-ideal conditions:

(13) $$\begin{array}{l} m\ddot{x}-2nKm\Omega \dot{y}+b_{x}\dot{x}+b_{xy}\dot{y}+k_{x}x+k_{xy}y=F_{x}\\ m\ddot{y}+2nKm\Omega \dot{x}+b_{y}\dot{y}+b_{xy}\dot{x}+k_{y}y+k_{xy}x=F_{y} \end{array}$$

where, *b_xy_* express the asymmetric damping of two electrode axes; *k_xy_* is the asymmetric stiffness coefficient of two electrode axes. Under the force balance mode, vibration in drive direction is controlled at resonance frequency. The vibration function is $x\left(t\right)=A\sin(\omega_{x}t)$. For ease of analysis, the electrostatic force applied to sense direction can be ignored. The vibration in the sense direction can be described as

(14) $$m\ddot{y}+b_{y}\dot{y}+k_{y}y=-\left(2nKm\Omega +b_{xy}\right)A\omega_{x}\cos\omega_{x}t-k_{xy}A\sin\omega_{x}t$$

The steady-state solution of Equation (14) is

(15) $$\begin{array}{ll} y\left(t\right) & =C_{1}\cos\omega_{x}t+C_{2}\sin\omega_{x}t\\ & =\frac{\left(\omega_{x}^{2}m-k_{y}\right)\left(2nKm\Omega +b_{xy}\right)\omega_{x}+k_{xy}b_{y}\omega_{x}}{{\left(\omega_{x}^{2}m-k_{y}\right)}^{2}+{\left(b_{y}\omega_{x}\right)}^{2}}A\cos\omega_{x}t\\ & +\frac{-\left(2nKm\Omega +b_{xy}\right)b_{y}\omega_{x}-k_{xy}\left(k_{y}-\omega_{x}^{2}m\right)}{{\left(\omega_{x}^{2}m-k_{y}\right)}^{2}+{\left(b_{y}\omega_{x}\right)}^{2}}A\sin\omega_{x}t \end{array}$$

If the resonator were ideal, there would be no frequency splitting, no asymmetric damping and asymmetric stiffness. Using the conversion $k_{yy}=\omega_{y}^{2}m$, Equation (15) is simplified as

(16) $$y\left(t\right)=\frac{-2nKm\Omega }{b_{y}\omega_{x}}A\sin\omega_{x}t$$

The Equation (16) shows that in an ideal DRG, the vibration’s amplitude in the sense direction is directly proportional to the input angular rate Ω, and its phase corresponds to the phase of vibration in the drive direction. Under force balance control, the force-to-rebalance loop eliminates this response. Therefore, Ω is obtained directly by demodulating the applied force of the force-to-rebalance loop.

Considering the accuracy of the practical process, fabrication errors introduced frequency splitting, and generated different resonance frequencies in the drive and sense directions. Equation (16) changed when frequency splitting exists. The asymmetric stiffness coefficient has the following relationship

(17) $$k_{xy}=\frac{\omega_{x}^{2}-\omega_{y}^{2}}{2}\sin2n\theta_{\omega }=\left(\omega_{x}^{2}-\omega_{y}^{2}\right)\sin n\theta_{\omega }\cos n\theta_{\omega }$$

where, *θ*_ω_ is the rigid frequency reduction axis as shown in Figure 2b

Substitute (17) into (15) gets

(18) $$\begin{array}{ll} y\left(t\right) & =C_{1}\cos\omega_{x}t+C_{2}\sin\omega_{x}t\\ & =\frac{\left(\omega_{x}^{2}-\omega_{y}^{2}\right)\left[2nKm^{2}\Omega +\sin n\theta_{\omega }\cos n\theta_{\omega }b_{y}\right]\omega_{x}}{{\left(\omega_{x}^{2}-\omega_{y}^{2}\right)}^{2}m^{2}+{\left(b_{y}\omega_{x}\right)}^{2}}A\cos\omega_{x}t\\ & +\frac{-2nKm\Omega b_{y}\omega_{x}-\left(\omega_{x}^{2}-\omega_{y}^{2}\right)\sin n\theta_{\omega }\cos n\theta_{\omega }m\left(\omega_{x}^{2}-\omega_{y}^{2}\right)}{{\left(\omega_{x}^{2}-\omega_{y}^{2}\right)}^{2}m^{2}+{\left(b_{y}\omega_{x}\right)}^{2}}A\sin\omega_{x}t\\ & =\frac{\left(\omega_{x}+\omega_{y}\right)\left[2nKm^{2}\Omega +\sin n\theta_{\omega }\cos n\theta_{\omega }b_{y}\right]}{{\left(\omega_{x}+\omega_{y}\right)}^{2}\Delta \omega^{2}m^{2}+{\left(b_{y}\omega_{x}\right)}^{2}}\Delta \omega A\omega_{x}\cos\omega_{x}t\\ & -\frac{2nKm\Omega b_{y}\omega_{x}+\sin n\theta_{\omega }\cos n\theta_{\omega }{\left(\omega_{x}+\omega_{y}\right)}^{2}\Delta \omega^{2}m}{{\left(\omega_{x}+\omega_{y}\right)}^{2}\Delta \omega^{2}m^{2}+{\left(b_{y}\omega_{x}\right)}^{2}}A\sin\omega_{x}t \end{array}$$

where, Δ*ω* is the difference between the resonance frequency in the drive and sense directions.

According to Equation (18), the main effects of frequency splitting on DRG output are listed as follows:

(1) Introduce an error signal orthogonal to the useful angular rate output;

(2) The form ${\left(\omega_{x}+\omega_{y}\right)}^{2}\Delta \omega^{2}m^{2}$ is in the denominator. It will greatly reduce the scale factor of DRG and decrease the sensitivity as a result;

(3) Equation (18) shows that the output angular rate information contains in-phase error caused by frequency splitting. This in-phase error brings zero drift to the gyroscope, and it can be represented as

(19) $$\Omega_{ERROR}=-\frac{2nKmb_{y}\omega_{x}\left(\Omega +\frac{{\left(\omega_{x}+\omega_{y}\right)}^{2}\Delta \omega^{2}m}{2nKmb_{y}\omega_{x}}\sin n\theta_{\omega }\cos n\theta_{\omega }\right)}{{\left(\omega_{x}+\omega_{y}\right)}^{2}\Delta \omega^{2}m^{2}+{\left(b_{y}\omega_{x}\right)}^{2}}A\sin\omega_{x}t$$

### 3.4. System Implementation Scheme

**Figure 5:**
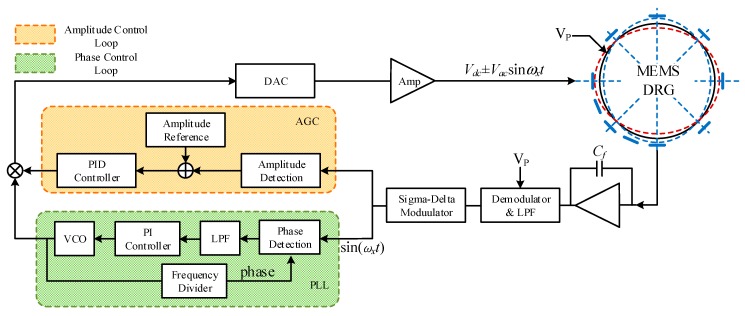
Schematic diagram of the frequency control loop and the amplitude control loop.

Figure 5 is the schematic diagram of the frequency control loop and the amplitude control loop. AGC loop and phase tracking loop are used to maintain the stability amplitude and phase of the resonator vibration. The AGC loop gets the amplitude of antinodes and adjusts it in real time through a proportion integration differentiation (PID) controller. The phase locking loop (PLL) loop compares the antinode signal with the return signal of the voltage-controlled oscillator (VCO) at the phase detector, the contrast result is input VCO by the proportion integration (PI) controller as error signal to change VCO output. The PLL loop not only generates the drive signal required by resonator, but also a reference demodulation signal for demodulation of the output signal to obtain the input information of the gyro’s angular velocity. 

**Figure 6:**
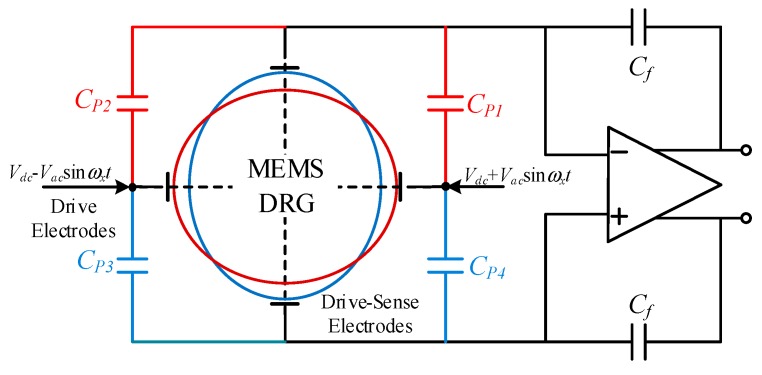
DRG drive signal coupled to drive-sense electrodes.

Due to the coupling of parasitic capacitances between drive electrodes and drive-sense electrodes, the sense electrodes are easily disturbed by the drive signal. As shown in Figure 6, CP1, CP2, CP3 and CP4 are coupling capacitances between differential electrodes. This interference will cause the phase deviation at the sense end of the gyroscope and make the gyroscope unable to reach self-excited vibration at the resonance frequency. This design loads the high-frequency carrier signal at the central anchor of the concentric rings. In this way, the drive signal can be modulated to high frequency and the interference near resonance frequency at the sense end reduced. 

The interference signal of amplifier output is

(20) $$V_{n}=V_{ac}\left(C_{P1}+C_{P3}-C_{P2}-C_{P4}\right)\sin\left(\omega_{x}t\right)/C_{f}$$

This signal phase is orthogonal to the sense signal in drive mode. The superposition of two signals results in the drive signal’s amplitude deviation and phase deviation. Loading the high-frequency carrier $V_{P}=V_{P0}\sin\left(\omega_{P}t\right)$ at the central anchor, the output of charge amplifier is

(21) $$V_{ch\arg e\_out}=-\frac{2N_{1}N_{2}\varepsilon^{2}y^{2}z^{2}QV_{P0}V_{dc}V_{ac}}{C_{f}x^{4}m\omega_{x}^{2}}G\sin\left(\omega_{p}t\right)\cos\left(\omega_{x}t\right).$$

Equation (21) shows that the drive-sense signal is modulated to a high frequency sideband signal, while the coupling signal is still at the drive frequency. The drive-sense signal can be restored through high frequency demodulation, and the coupling signal can be eliminated by a low pass filter. In this way, the phase of the closed self-excited loop can be guaranteed and the low frequency noise is suppressed.

**Figure 7:**
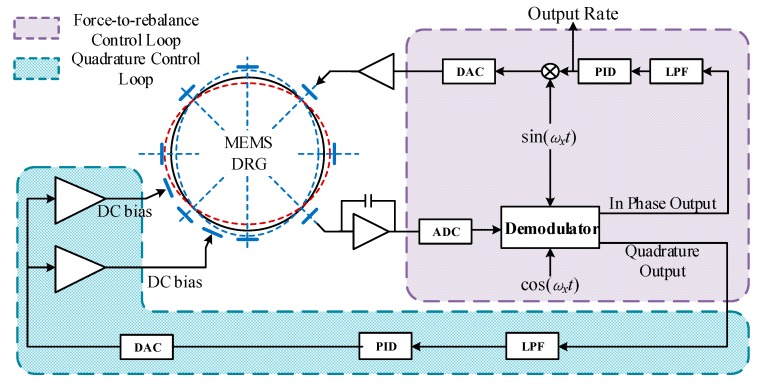
Schematic diagram of force-to-rebalance control loop and quadrature control loop.

Figure 7 gives the schematic diagram of the force-to-rebalance control loop and quadrature control loop. Under force balance mode, the vibration shape of the resonator remains non-precession state under the action of external forces; the excitation force on the force-balance-control electrode is proportional to the input angular velocity. From this, the angular velocity of the MEMS DRG can be calculated according to the applied feedback force. The force-to-rebalance loop reads out the output signal of wave nodes and demodulates it. Demodulation gives in-phase signal and quadrature signal parts. The in-phase signal passes through the low-pass filter (LPF) and PID controller back to the detection electrode, and keeps the vibration mode of the resonator stationary relative to the shell. The quadrature signal feeds back into the quadrature loop to generate the error signal, which is applied to the mode control electrode to eliminate frequency splitting.

## 4. Circuit Implementation Details

### 4.1. Weak Signal Detection Circuit

**Figure 8:**
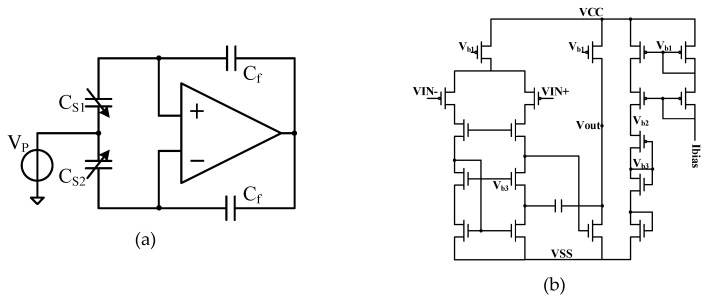
(**a**) Charge amplifier based on high frequency carrier principle, (**b**) topology of nested Miller Compensation second-order operational amplifier.

According to the DRG sense element, the electrical signal changes caused by the input angular velocity can be equivalent to the charge changes. Therefore, the charge amplifier is used in the drive loop and sense loop to convert the charge transfer into voltage signal. The charge amplifier based on the high-frequency carrier principle is shown in Figure 8a. In the sense direction, the displacement of the harmonic oscillator is extraordinary small. The current changes generated at sense electrodes are slight when voltage is applied to the mass. Displacement at the sense end with input angular velocity of $\Omega =\Omega_{0}\cos\omega_{i}t$ can be expressed as

(22) $$y\left(t\right)=B_{1}\Omega_{0}\cos\left(\omega_{i}t\right)\sin\left(\omega_{x}t\right)+B_{2}\Omega_{0}\sin\left(\omega_{i}t\right)\cos\left(\omega_{x}t\right),$$

where, *B*_1_ and *B*_2_ are parameters related to the sensitive structure. According to the principle shown in Figure 8a, the output of charge amplifier is

(23) $$V_{ch\arg e}\left(t\right)=\frac{2K_{X/C}}{C_{f}}y(t)\sin\left(\omega_{p}t\right),$$

where, *K_X/C_* is the conversion coefficient between displacement and capacitance.

To achieve high performance of the charge amplifier, a secondary operational amplifier was used, the topological structure is shown in Figure 8b. To realize the purpose of high speed, low noise, low offset, low temperature drift and high gain, a telescopic structure was used for the first level. For the second level, we adopted a common source output to improve the output range. Miller compensation ensured the phase margin and improved the closed-loop stability; it also effectively reduced the output impedance of the amplifier. The PMOS (P-channel Metal Oxide Semiconductor) transistors’ aspect ratios were designed reasonably to reduce the low frequency 1/f noise and thermal noise.

### 4.2. Design of ΣΔ the Analog to Digital Converter (ADC)

The ADC is an indispensable part of the DRG digital interface circuits. The mechanical structure detects and outputs the analog signal. In digital interface circuit design, an analog signal need to be digitized by ADC circuits. The ADC’s precision affects the performance of the DRG system directly. This design used a switched-capacitor (SC) ΣΔ modulator to achieve signal conversion. Compared with a successive approximation register (SAR) structure, the ΣΔ structure has higher conversion accuracy. By using oversampling and noise-shaping technologies, the white noise in the passband is transformed to high frequency, which effectively reduces the noise floor in the passband and achieves higher detection accuracy. One-bit digital output eliminates nonlinearity interference. The bandwidth requirement of ADC in drive circuit is no less than 10kHz, SNR no less than 100dB. A third-order CIFF (cascade-of-integrators, feedforward) ΣΔ modulator is presented in this design to achieve analog-to-digital conversion.

**Figure 9:**
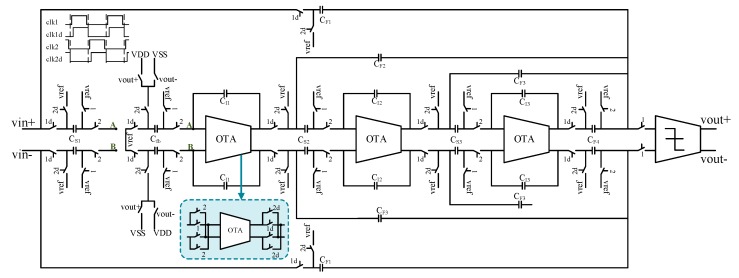
Circuit design of cascade-of-integrators, feedforward (CIFF) ΣΔ analog modulator.

The circuit design of the CIFF ΣΔ analog modulator is shown in Figure 9. The function of the integrator was realized by using an SC circuit. The fully differential design can effectively improve the SNR and suppress the second harmonic distortion. In the last stage of the circuit, the non-delay switched-capacitor structure was used for summation to avoid the use of an analog adder. In order to reduce the low frequency noise, adopted the chopper technology at the input end of the first operational amplifier. The ΣΔ modulator completed the filter as well as the ADC functions at the same time, which facilitates subsequent signal processing.

According to Figure 9, the signal transfer function (STF) and noise transfer function (NTF) of the modulator can be obtained as

(24) $$STF=\frac{(\frac{C_{F4}}{\sum_{i=1,2,3,4}C_{Fi}}\prod_{i=1,2,3}\frac{C_{Si}}{C_{1i}})H^{3}+(\frac{C_{F3}}{\sum_{i=1,2,3,4}C_{Fi}}\prod_{i=1,2}\frac{C_{Si}}{C_{1i}})H^{2}+\frac{C_{F2}}{\sum_{i=1,2,3,4}C_{Fi}}\frac{C_{S1}}{C_{11}}H+\frac{C_{F1}}{\sum_{i=1,2,3,4}C_{Fi}}}{(\frac{C_{fb}}{C_{11}}\frac{C_{F4}}{\sum_{i=1,2,3,4}C_{Fi}}\prod_{i=2,3}\frac{C_{Si}}{C_{1i}})H^{3}+(\frac{C_{fb}}{C_{11}}\frac{C_{S2}}{C_{12}}\frac{C_{F3}}{\sum_{i=1,2,3,4}C_{Fi}})H^{2}+\frac{C_{fb}}{C_{11}}\frac{C_{F2}}{\sum_{i=1,2,3,4}C_{Fi}}H+1},$$

(25) $$NTF=\frac{1}{(\frac{C_{fb}}{C_{11}}\frac{C_{F4}}{\sum_{i=1,2,3,4}C_{Fi}}\prod_{i=2,3}\frac{C_{Si}}{C_{1i}})H^{3}+(\frac{C_{fb}}{C_{11}}\frac{C_{S2}}{C_{12}}\frac{C_{F3}}{\sum_{i=1,2,3,4}C_{Fi}})H^{2}+\frac{C_{fb}}{C_{11}}\frac{C_{F2}}{\sum_{i=1,2,3,4}C_{Fi}}H+1}.$$

It can be seen from the above equations that STF and NTF decrease, and the signal energy at the input end of the quantizer reduces when the feedback capacitance C*_fb_* increases. In this design, the feedback capacitances are separated from the sampling capacitances. The stability and input range of the modulator enhance when the feedback capacitance increases; this can achieve the large input signal swing.

**Figure 10:**
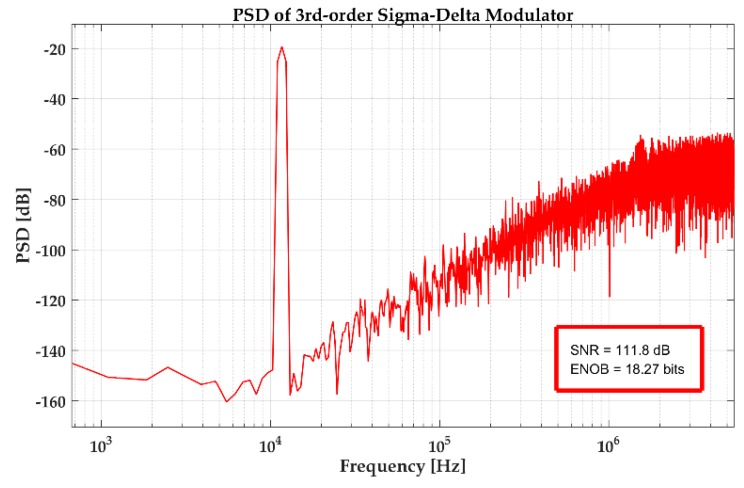
Power spectral density (PSD) of the proposed ΣΔ modulator.

Figure 10 gives the power spectral density (PSD) of the ΣΔ modulator. The SNR is 111.8 dB with 11.7 kHz input signal, and the frequency of the sampling clock is 2 MHz. The noise floor is lower than ‒140 dB. These results satisfied the design requirements. 

## 5. Experimental Results and Discussion

**Figure 11:**
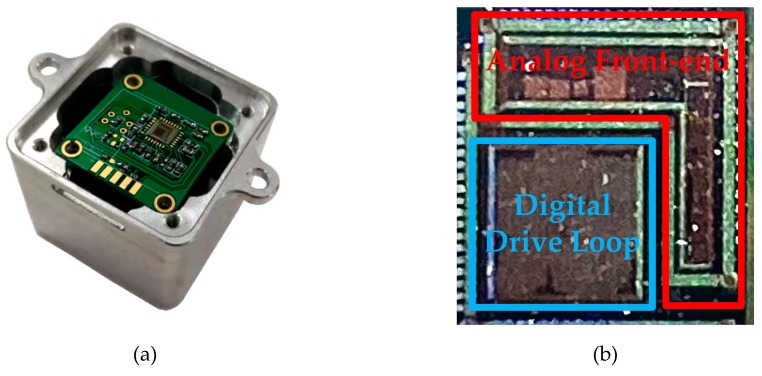
(**a**) Prototype test package, (**b**) layout of the overall chip.

To verify the proposed closed-loop MEMS digital DRG system design, the corresponding test circuit was built. The analog circuit ASIC for MEMS DRG gyroscopes is fabricated in a standard 0.35 μm CMOS BCD process, and occupies a 25 mm^2^ (5 × 5 mm) area. Figure 11a shows the prototype gyroscope, Figure 11b shows the layout the overall chip. The vacuum packaged MEMS sensing element, the analog ASIC as well as the PCB testing-board were encapsulated in an aluminum cube shell with 3 cm side length. In order to save volume in the prototype, the sensing element was fixed back-to-back with the testing-board. In order to ensure the accuracy of this design, FPGA was used to verify the digital circuit. All tests were conducted under a normal temperature of 25 °C. The whole system runs from a 5 V supply and consumes a current of 50 mA.

**Figure 12:**
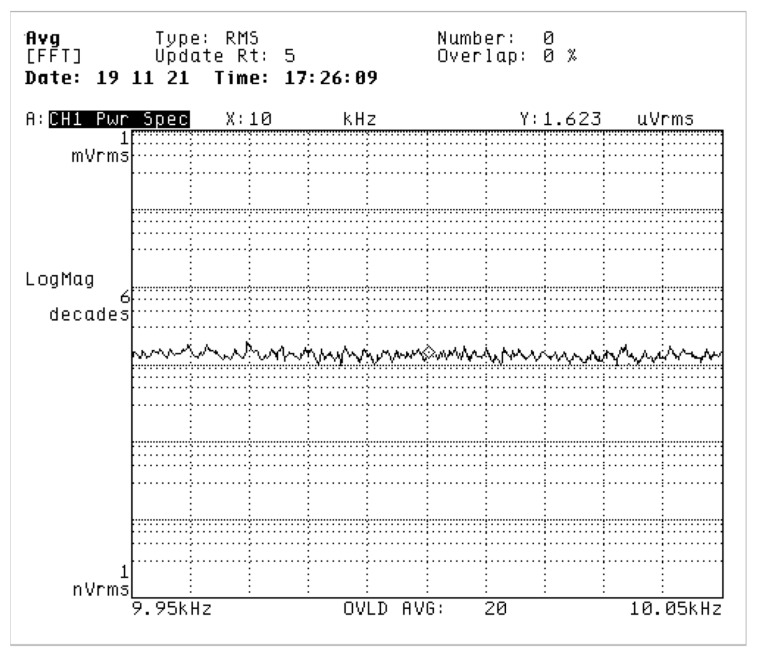
Output noise of pre-stage charge amplifier.

Firstly, the noise level of the pre-stage charge amplifier output designed in this work is tested. The output voltage noise density within the ± 50 Hz bandwidth at a frequency of 10 kHz was measured by a dynamic spectrum analyzer. From the test results shown in Figure 12, the output voltage noise of the charge amplifier reaches 1.623 μV/√Hz.

**Figure 13:**
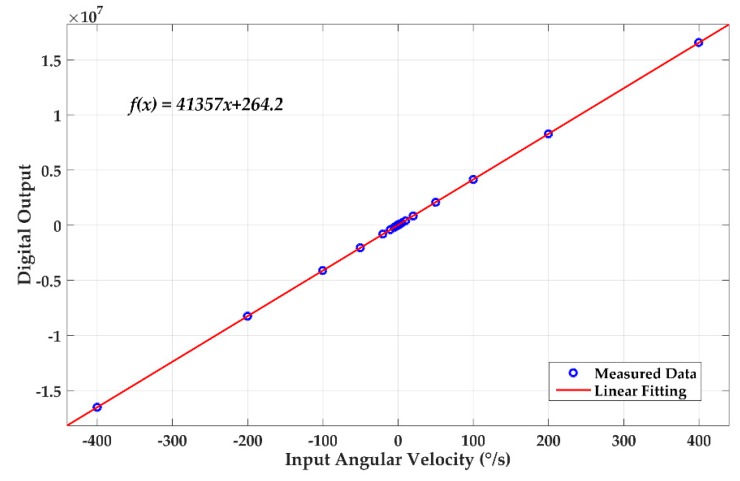
DC transfer function after calibration.

The scale-factor is the basis of parameter representation in the gyroscope system. The scale factor can be measured simultaneously with the output linearity. The DRG was placed on a turntable and we applied an angular velocity signal within ± 400 °/s and measured the corresponding output digital signal. Figure 13 shows the measured data and the linear fitting curve. By converting the digital output into voltage, the scale-factor can be obtained as 6.2 mV/°/s with a zero-rate output of 40 µV.

**Figure 14:**
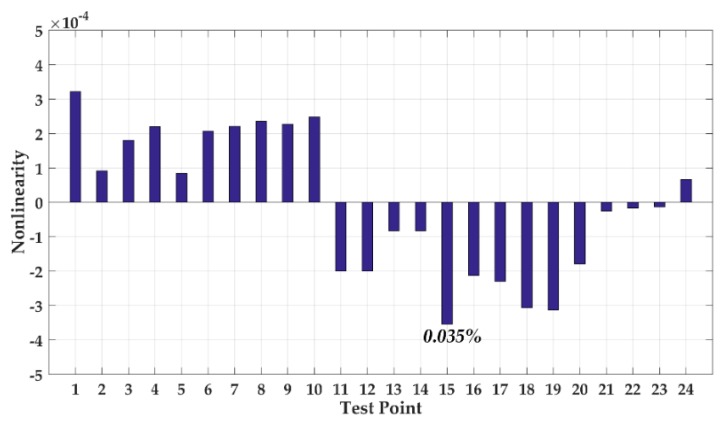
Nonlinearity test result of DRG.

We processed the obtained data, and the nonlinearity test result is shown in Figure 14. The fitting nonlinearity over the measurement range reaches up to 0.035%.

**Figure 15:**
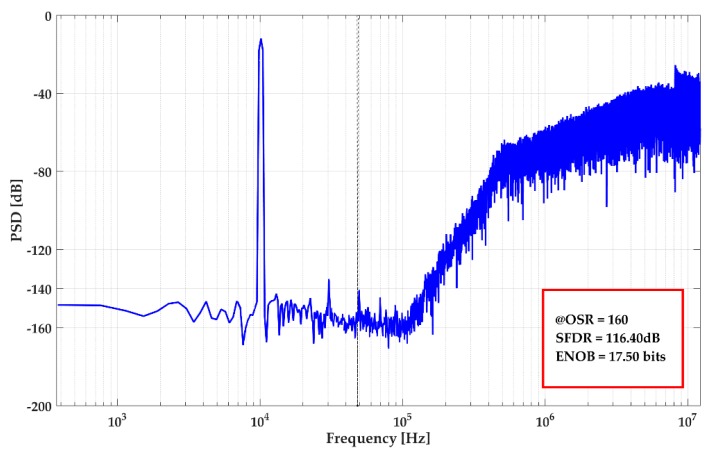
Harmonic test result of DRG.

The harmonic test result is given in Figure 15. Under the oversampling rate (OSR) of 160, the spurious-free dynamic range (SFDR) is 116.4 dB with 17.5 bits ENOB (Effective Number of Bits). The third-harmonic is ‒135.2 dB.

**Figure 16:**
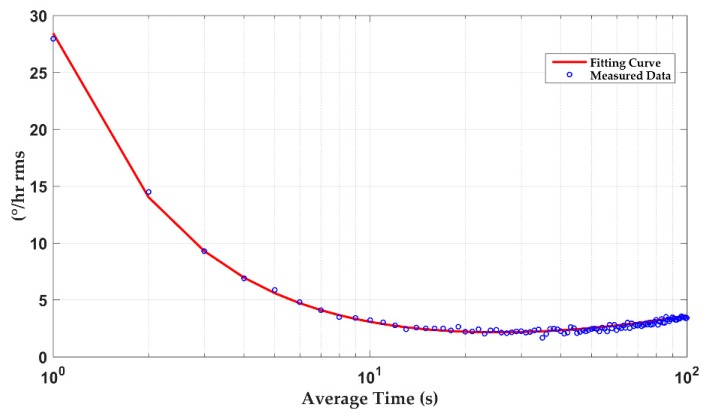
Standard Allen variance at 25 °C.

The influence of external vibration and temperature drift on the DRG’s detection accuracy need to be avoided during stability tests. The DRG was placed in a constant temperature box at 25 °C and the turntable rotated at a fixed angular velocity. Figure 16 gives the tested DRG’s standard Allen variance. This index includes various noise sources and represents the long-term stability of the system. Experimental results show a bias instability of 2.1 °/h, which satisfies many applications.

The above experimental results meet the design expectation. Comparing the experimental results with prior works as shown in Table 2. Research on DRGs mainly focuses on the sensing elements; there are few studies on control circuit schemes. Therefore, less attention is paid to circuit properties (e.g. input range, nonlinearity, power consumption and chip area). This work uses the similar circuit realization manner from [22] but obtained better properties. Ref. [23] used PCD and FPGA to verify DRG function without the consideration of circuit integration, and the nonlinearity of [23] is higher than this work. [24] focused on the open-loop detection interface ASIC based on the vibratory gyroscope, with less control loops, an it achieved monolithic integration. The comparison with [24] shows the circuit properties directly of this work achieves the same performance level as the vibratory gyroscopes.

It is also worthwhile to mention further performance improvement. In terms of performance indicators, the nonlinearity of this system can be reduced through nonlinear drive control technology. For higher integration application requirements, ΣΔ modulators can use time division multiplexing technology to reduce the analog layout area. As the increasing of the DRG quality is a factor, the resulting problem of system stability needs to take into consideration. To achieve higher sense accuracy, the system also needs to be compensated. It is necessary to establish a DRG temperature drift model and carry out compensation technology research. From the perspective of high integration and high precision demanded in spatial navigation, the DRG ASIC is a positive development direction.

## 6. Conclusions

In this paper, a digital closed-loop sense MEMS DRG circuit design based on an integrated analog front-end is provided. The system adopted includes a force balance mode, realized digital closed-loop drive and closed-loop sense. This work makes contributions to the study of DRG integrated circuits, system theory research and circuit implementation details. This article gives the motion regulation of DRG sensing elements, discusses the closed-loop self-excited condition and studies the system output influenced by frequency splitting. The use of a high-frequency carrier ensured the stability of the self-excited drive. Closed-loop sense schemes can obtain higher detection accuracy compared with open loop. This design realized the on-chip integration of analog front-end and digital drive circuit, which reduced the area of the circuit and implemented the circuit function completely. 

The integrated circuit was manufactured in a standard 0.35 μm CMOS technology. Experimental results show a full-scale range of ± 400°/s, 116.4 dB SFDR of system output and a bias instability of 2.1 °/h with nonlinearity of 0.035%.

## Figures and Tables

**Table 1:** MEMS DRG performance comparisons of different working modes.

Key Indicators	Full Angle Excitation Mode (Open-Loop)	Force Balance Mode (Closed-Loop)
Detect parameter	Angle detect	Angle rate detect
Application	High rotate speed	Low rotate speed
Measure accuracy	Difficult to achieve high precision	High precision
Influence of structure errors	large	small

**Table 2:** Properties summary and comparison.

Properties	[22]	[23]	[24]	This Work
Input range	-	-	± 400 °/s	± 400 °/s
scale-factor	55 μV/°/s	-	5.5 mV/◦/s	6.2 mV/°/s
Nonlinearity	-	± 0.15%	0.016%	0.035%
Standard Allen variance (25 °C)	20 °/h	1.5 °/h	2.2 °/h	2.1 °/h
Circuit realization	Integrated analog front-end	PCB and FPGA	ASIC	Integrated analog front-end and integrated drive loop
